# Smallest detectable change in volume differs between mass flow sensor and pneumotachograph

**DOI:** 10.1186/1756-0500-4-23

**Published:** 2011-01-28

**Authors:** Herman Groepenhoff, Caroline B Terwee, Patrick MC Jak, Anton Vonk-Noordegraaf

**Affiliations:** 1 Department of pulmonology and institute for Cardiovascular Research (ICaR-Vu) VU University Medical Center, Amsterdam, 1007 MB, The Netherlands; 2 Department of Epidemiology and Biostatistics and the EMGO Institute for Health and Care Research. VU University Medical Center, Amsterdam, 1007 MB, The Netherlands

## Abstract

**Background:**

To assess a pulmonary function change over time the mass flow sensor and the pneumotachograph are widely used in commercially available instruments. However, the smallest detectable change for both devices has never been compared. Therefore, the aim of this study is to determine the smallest detectable change in vital capacity (VC) and single-breath diffusion parameters measured by mass flow sensor and or pneumotachograph.

**Method:**

In 28 healthy pulmonary function technicians VC, transfer factor for carbon monoxide (DLCO) and alveolar volume (VA) was repeatedly (10×) measured. The smallest detectable change was calculated by 1.96 x Standard Error of Measurement ×√2.

**Findings:**

The mean (range) of the smallest detectable change measured by mass flow sensor and pneumotachograph respectively, were for VC (in Liter): 0.53 (0.46-0.65); 0.25 (0.17-0.36) (*p *= 0.04), DLCO (in mmol*kPa^-1^*min^-1^): 1.53 (1.26-1.7); 1.18 (0.84-1.39) (*p *= 0.07), VA (in Liter): 0.66. (0.53-0.82); 0.43 (0.34-0.53) (*p *= 0.04) and DLCO/VA (in mmol*kPa^-1^*min^-1^*L^-1^): 0.22 (0.19-0.28); 0.19 (0.14-0.22) (*p *= 0.79).

**Conclusions:**

Smallest detectable significant change in VC and VA as measured by pneumotachograph are smaller than by mass flow sensor. Therefore, the pneumotachograph is the preferred instrument to estimate lung volume change over time in individual patients.

## Background

To measure pulmonary function changes over time the mass flow sensor and the pneumotachograph are widely used instruments. Due to international equipment requirements, calibration, validation and measurement procedures both measurement devices are assumed to have identical reliability[1,2]. However the smallest detectable change, which is the smallest significant change that can be detected between individual measurements, has in neither device, been determined. The smallest detectable change is a very useful parameter for clinical practice because it shows which changes in a single patient can be considered a 'real' change. Hence, pulmonary function instruments with the smallest detectable change are best suited for evaluating changes as a result of disease progress or applied therapy.

The aim of this study is to determine the smallest detectable change of vital capacity (VC) and single-breath diffusion parameters measured by mass flow sensor and pneumotachograph.

## Materials and methods

### Study design

A total of 28 (two to six per hospital) healthy, non-smoking pulmonary function technicians from eight different Dutch hospitals measured repeatedly (10 times) their VC and single-breath diffusion parameters (transfer factor for carbon monoxide (DLCO) and alveolar volume (VA). Each hospital had three different pulmonary function apparatus of a single type that were used in a random sequence, as part of the standard biological calibration procedure. In three hospitals, eight persons measured their pulmonary function by mass flow sensor (Vmax series, Sensor medics, Yorba Linda, USA). In the other five hospitals, 20 persons measured their pulmonary function by pneumotachograph (Masterlab series, Jeager, Wurzburg, Germany). All measurements were performed within a time frame of six months between October 2008 and September 2009. All pulmonary function tests were performed after a careful calibration procedure of the instruments according to manufacturer's instructions. Pulmonary function tests were performed according to ERS guidelines. Briefly, for the VC, the largest value of at least three technically satisfactory determinations not exceeding the next highest one by more than 150 ml was used[1]. For DLCO measurements, the mean of at least two technically satisfactory DLCO determinations (maximal difference ≤10% of highest value) was taken.

Ethical approval and informed consent was not required because this biological validation study does not fall under the Dutch law on Human Research (WMO). This was approved by the Medical Ethics Committee of the VU University Medical Center.

### Statistical analyses

Results were expressed as mean ± standard deviation or range. Unpaired t-test was used to compare pulmonary function measurement results between laboratories using a mass flow sensor and a pneumotachograph. Coefficient of variation was calculated for each pulmonary function parameter for each individual as the standard deviation divided by the mean of ten repeated measurements. The variance components were estimated by two-way analysis of variation with SPSS (version 15.0) with apparatus and occasion as random factors, using the restricted maximum likelihood method. From these variance components, the standard error of measurement was calculated by taking the square root of the sum of apparatus, occasion and random error variance. (SEM = √(σ^2 ^apparatus + σ^2 ^occasions +σ^2 ^error)). The smallest detectable change was calculated as 1.96 × SEM × √2[3]. Coefficient of variation, standard error of measurement, different sources of measurement variation (between apparatuses, occasions, random) and smallest detectable change differences between the mass flow sensor and pneumotachograph measurements for all pulmonary function parameters were checked by Mann Whitney *U *test. In all analyses *p *< 0.05 was considered significant.

## Results

As expected in this healthy study population, the subjects showed absolute pulmonary function results within the normal physiological range[4,5]. Furthermore, mean pulmonary function outcome results measured by mass flow sensor were not different from pneumotachograph measurements (Table 1).

**Table 1:** Pulmonary function results on three different pulmonary function instruments.

	MF	PT	*p*
**Number of hospitals**	3	5	
**Number of subjects**	8	20	
**VC (L.)**	5.0 (0.7)	4.7 (1.2)	*0.47*
**DLCO (mmol/kPa/min)**	9.9 (2.3)	9.0 (2.4)	*0.41*
**VA (L.)**	6.4 (1.0)	5.8 (1.4)	*0.29*
**DLCO/VA (mmol/kPa/min/L.)**	1.5 (0.3)	1.6 (0.2)	*0.81*

Results are expressed as mean and standard deviation. Mass flow sensor (MF); pneumotachograph (PT); VC: vital capacity; DLCO: transfer factor for carbon monoxide; VA: alveolar volume.

**Figure 1:**
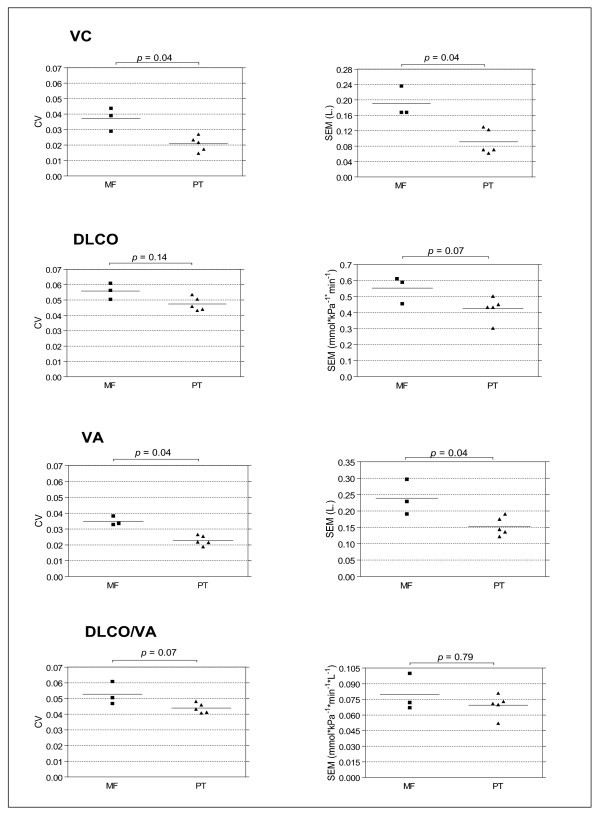
**Coefficient of variation (CV, left) and standard error of measurement (SEM, right) of vital capacity (VC), transfer factor for carbon monoxide (DLCO), alveolar volume (VA) and DLCO/VA**. Each dot represents one hospital. MF is mass flow sensor; PT is pneumotachograph.

Figure 1 shows that the coefficient of variation and the standard error of measurement for VC and all single-breath diffusion parameters measured by mass flow sensor were higher than those calculated from the pneumotachograph measurements. However, significance was reached for VC and VA differences only.

**Figure 2:**
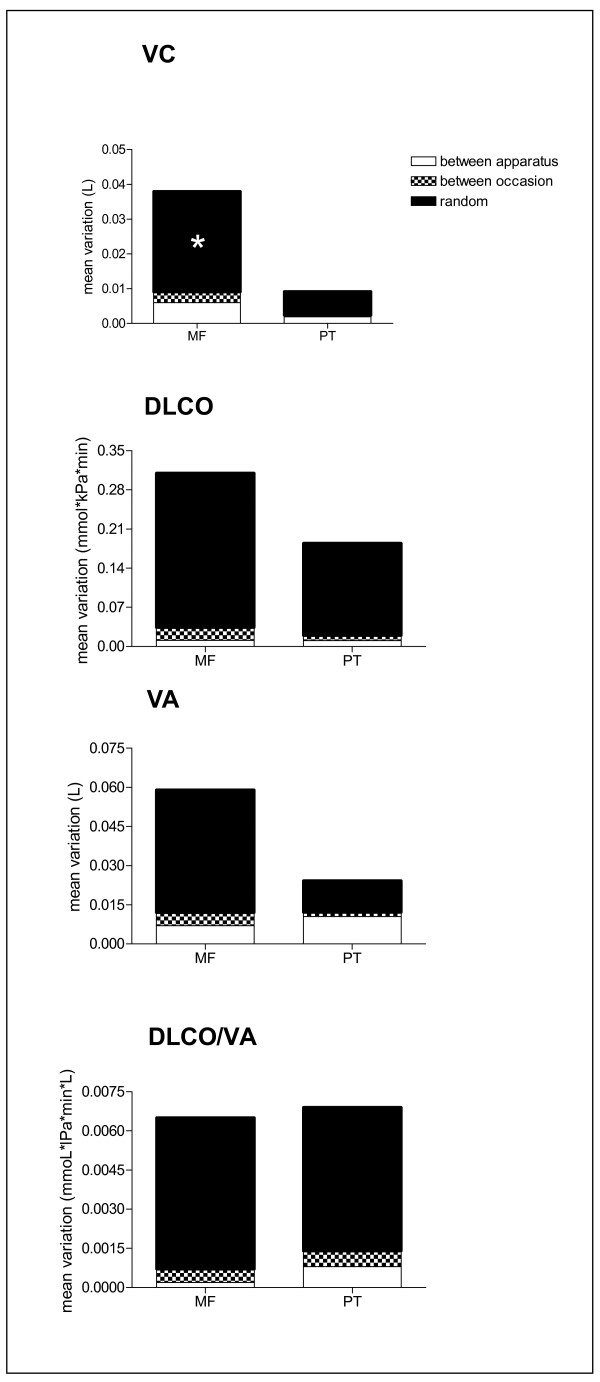
**Total error variance (= between apparatus, between occasion and random variation) for vital capacity (VC), transfer factor for carbon monoxide (DLCO), alveolar volume and DLCO/VA measured on three apparatuses**. * *p *< 0.05 for random variation between mass flow sensor (MF) and pneumotachograph (PT) measurements.

Random error is the major source of error variation in all pulmonary function results for both measurement devices, followed by (between) apparatus variation. Except for DLCO and DLCO/VA measured by mass flow sensor were the variation due to (between) occasion was slightly higher than between apparatus variation (Figure 2).

### Smallest detectable change

Smallest detectable change for all pulmonary function parameters measured by pneumotachograph were lower compared to the mass flow sensor measurements. As was the case with the standard error of measurement, statistical significance was reached for VC and VA only (Table 2).

**Table 2:** Smallest detectable change measured by mass flow sensor (MF) or pneumotachograph(PT).

	MF	PT	*p*
**VC (L.)**	0.53 (0.46 - 0.65)	0.25 (0.17 - 0.36)	0.04
**DLCO (mmol/kPa/min**	1.53 (1.26 - 1.70)	1.18 (0.84 -1.39)	0.07
**VA (L.)**	0.66 (0.53 -0.82)	0.43 (0.34 - 0.53)	0.04
**DLCO/VA (mmol/kPa/min/L.)**	0.22 (0.19 - 0.28)	0.19 (0.14 - 0.22)	0.79

Results are expressed as mean and range. Mass flow sensor (MF); pneumotachograph (PT); VC: vital capacity; DLCO: transfer factor for carbon monoxide; VA: alveolar volume

## Conclusions and Discussion

In this multi-centre study we found that in a realistic clinical setting the smallest detectable change for the volume measurements, VC and VA, was smaller when measured by pneumotachograph than by mass flow sensor. A similar result was found for the smallest detectable difference for DLCO, but did not reached statistical significance.

These differences were mainly due to a lower measurement error (lower variance, Figure 2) of the pneumotachograph measurements. The measurement error in this study was reflected in absolute values by the standard error of measurement as well as the coefficient of variation. The coefficient of variation values in this study were similar to the values found in long term repeatability measurements in healthy subjects reported by Pennock et al. [6] and Jensen et al. [7] These studies showed that the measurement error of the simple VC assessment was smaller than the measurement error of the much more comprehensive single breath diffusion measurement. Our study confirmed these findings.

Although reliability of pulmonary function tests is commonly estimated by the coefficient of variation Hankinson et al. recommend expressing pulmonary function test variability in absolute terms [8]. The coefficient of variation provides information about the measurement error related to the mean value of a sample of repeated measurements over time. This mean value of one individual patient is usually not known, as in clinical practice usually one single measurement for an individual is available. For that reason the smallest detectable change, which is important for clinical decision making, cannot be estimated by the coefficient of variation. Nonetheless, from our study it can be seen that a coefficient of variation difference for VC of 1.6 percent between the measurent devices (Figure 1) doubles smallest detectable change from 0.25L to 0.53L (table 2). This means for clinical practice that a difference between two consecutive VC measurements in time smaller than 250 ml measured by pneumotachgraph and smaller than 530 ml when measured by a mass flow sensor can not be distinguished from measurement error.

An additional important advantage of the standard error of measurement (Figure 2) is the possibility to analyze different sources of error variance (between-apparatus, between occasion and random). This knowledge provides direct information of the main cause of error. Quality control management of a pulmonary function laboratory should, when possible, act on this knowledge[9]. In case of a large between-apparatus variation one should investigate and solve the cause of this undesirable systematic difference. Our results show that for all pulmonary function parameters random variation is the main source of error for both measurement devices, with the largest values in the mass flow sensor. Other errors are relatively small. Consequently, total measurement variation, which is the sum of all error sources, for all pulmonary function parameters is, except for the DLCO/VA, are higher in the mass flow compared to the pneumotachograph data. Random variation can be due to (subject- biological, coincidence (or circumstance) and unsystematic instrument error. Since there are no differences in the way the pulmonary function tests are performed on both devices, we expect the biological and circumstance variations to be comparable between both devices. Thus, unsystematic error differences between the measurement devices seem to be the most likely explanation for the difference in random error between the mass flow sensor and pneumotachograph measurements. An increased between apparatus variation for the VC found in the mass flow sensor measurements suggests a higher systematic difference between the mass flow sensor compared to the between pneumotachograph apparatuses. Increased occasion variation points to a lower degree of repeatability for VC and DLCO of mass flow devices, compared to the pneumotachograph devices. It is unlikely that the differences in measurement error for the pulmonary function parameters between mass flow and pneumotachograph measurements are due to differences in absolute values because mean outcome results from the three "mass flow sensor" hospitals were not different from the values measured in the five "pneumotachograph" hospitals.

In clinical practice knowledge of the smallest detectable change in patients would be most informative. Apparently, this knowledge is difficult to obtain because one needs repeated measurements from long term, exceptionally stable, patients. Nonetheless, Pennock et al. showed a larger coefficient of variation in healthy subjects compared to patients in pulmonary function measurements[6]. Therefore, we speculate that the smallest detectable difference in patients is at least as large as the values estimated by the healthy subjects in this study.

In summary our results show that the total measurement error of one single VC or VA measured by pneumotachograph is lower compared to these measurements obtained by mass flow sensor. Consequently, the smallest detectable significant change between individual VC and VA measured by pneumotachograph are smaller than when measured by mass flow sensor measurements. Therefore, the pneumotachograph is the preferred instrument to estimate lung volume change over time in the individual patient.

Although pneumotach performs better than the mass flow sensor in terms of smallest detectable difference, the latter has the advantage that it can be used during exercise and is probably less influenced by temperature or pressure.

## Competing interests

The authors declare that they have no competing interests.

## Authors' contributions

HG participated in the design of the study, performed the statistical analysis and drafted the manuscript. CT helped to perform the statistical analysis and drafted the manuscript. PJ read the manuscript carefully which resulted in important feedback. AV participated in its design and helped to draft the manuscript. All authors read and approved the final manuscript.
